# Regional and Racial Differences in Smoking and Exposure to Secondhand Smoke: The Reasons for Geographic and Racial Differences in Stroke (REGARDS) Study

**Published:** 2011-08-15

**Authors:** Leslie A. McClure, Heather L. Murphy, Jeffrey Roseman, George Howard, Ann Malarcher

**Affiliations:** University of Alabama at Birmingham; University of Alabama at Birmingham, Birmingham, Alabama; University of Alabama at Birmingham, Birmingham, Alabama; University of Alabama at Birmingham, Birmingham, Alabama; Centers for Disease Control and Prevention, Atlanta, Georgia

## Abstract

**Introduction:**

Stroke mortality rates differ by race and region, and smoking and exposure to secondhand smoke are associated with stroke. We evaluated regional and racial differences in current smoking and secondhand smoke exposure among participants in the Reasons for Geographic and Racial Differences in Stroke (REGARDS) study.

**Methods:**

African American and white adults (n = 26,373) aged 45 years or older were recruited during 2003 through 2007. Logistic regression was used to examine the likelihood of current smoking and secondhand smoke exposure by race (African American vs white) and region. We compared the buckle of the stroke belt (the coastal plain region of North Carolina, South Carolina, and Georgia) with the stroke belt (the remainder of North Carolina, South Carolina, and Georgia, plus Alabama, Mississippi, Tennessee, Arkansas, and Louisiana) and compared each of these regions with the remaining contiguous states.

**Results:**

Among whites, no regional differences in current smoking were seen, but among African Americans, the odds of current smoking were 5% lower in the stroke belt, and 24% lower in the stroke buckle than those in the nonbelt region. Similarly, among whites no regional differences in exposure to secondhand smoke were found, whereas among African Americans, the odds of being exposed to secondhand smoke were 14% lower in the stroke buckle than for nonbelt residents.

**Conclusions:**

These data suggest that rates of current smoking and secondhand smoke exposure are not higher in regions that have higher stroke mortality and therefore cannot contribute to geographic disparities; nevertheless, given that 15% of our participants reported current smoking and 16% reported secondhand smoke exposure, continued implementation of tobacco control policies is needed.

## Introduction

Cigarette smoking is the leading preventable cause of death in the United States (1). Exposure to secondhand smoke (SHS) also causes detrimental health effects among both adults and children (2). Cigarette smoking is a major contributor to stroke and other cardiovascular diseases, approximately doubling the risk of ischemic stroke, increasing the risk of hemorrhagic stroke by 2 to 4 times (3,4), and contributing to 12% to 14% of all stroke deaths (5,6). Quitting smoking appears to reduce the risk of stroke mortality (5), and longer times since quitting are associated with lower stroke risk (7).

SHS exposure has been causally associated with coronary heart disease illness and death among nonsmoking adults, suggesting a biologically plausible association with stroke (2). The odds of incident stroke are approximately 30% to 80% higher among those exposed to SHS compared with those not exposed (7-9); exposure to SHS is a risk factor for both 3-year (10) and long-term progression (11) of intimal-medial thickness of the carotid artery.

Regional variations in smoking and SHS exposure have been hypothesized to contribute to high stroke mortality in the stroke belt, a region in the Southeast United States. An even higher rate of stroke mortality is found in the coastal plains of North Carolina, South Carolina and Georgia, known as the stroke buckle (10,12,13). The magnitude of the increased risk of stroke mortality associated with the stroke belt is similar for men and women but is approximately 20% greater for African Americans than for whites (14). Although smoking and exposure to secondhand smoke have been shown to be independent risk factors for cardiovascular diseases, including stroke, whether they could contribute to regional and racial disparities in stroke mortality is unknown.

In 1996, the National Cancer Institute published state-specific rates of smoking based on the Tobacco Use Supplement to the 1992-93 Current Population Survey, finding the highest smoking rates for whites in the South (supporting a contribution to regional disparities in stroke). The highest rates for African Americans were in the Midwest (15). Regional differences in smoking are also described by the National Survey on Drug Use and Health (NSDUH), which is conducted each year by the Substance Abuse and Mental Health Services Administration (16). Among 44,467 respondents aged 18 or older, 27.5% reported current smoking. Although these data indicate similar smoking prevalence for whites (27.9%) and African Americans (28.0%), there were regional differences. The highest smoking rates were in the East-South-Central United States and the lowest were in the Pacific region. Further, data from NSDUH indicate that the proportion of smokers is higher in rural than in urban areas (16). Updated data from the 2005 survey provide similar results (17). Additionally, among 22,990 respondents to the 2007 National Health Interview Survey, rates of smoking among men were marginally higher for African Americans than for whites, whereas rates for white and African American women were similar (18).

The reporting of these regional differences in current smoking follow census definitions of regions, where the "South" includes a number of states not included in the stroke belt (eg, Virginia, Florida, Texas), making the assessment of the association of geographic variations in stroke risk and smoking prevalence problematic to reconcile. To our knowledge, few available data report regional estimates of SHS exposure for the stroke belt and buckle regions rather than by state or larger regional areas, although limited data suggest that more African Americans than whites are exposed to SHS in the home (19). The objective of this study was twofold: first, to describe both racial and geographic variation in smoking behavior using boundaries aligned with stroke risk (eg, stroke belt, buckle of the stroke belt, and the rest of the nation), and second, to provide new data on racial and geographic variations in exposure to SHS.

## Methods

The Reasons for Geographic and Racial Differences in Stroke (REGARDS) study is a national population-based cohort study that recruited 30,229 participants aged 45 or older, of whom 55% were women, 42% were African American, and 58% were white. Recruitment of the cohort began in February 2003 and was completed in October 2007. We recruited 21% of the cohort from the buckle of the stroke belt (coastal plain region of North Carolina, South Carolina, and Georgia), 35% from the stroke belt states (the remainder of North Carolina, South Carolina, and Georgia, plus Alabama, Mississippi, Tennessee, Arkansas, and Louisiana), and the remaining 44% from the other 40 contiguous states. Exclusion criteria were race/ethnicity other than non-Hispanic African American or white, actively being treated for cancer, medical conditions preventing long-term participation in the study, cognitive impairment judged by the telephone interviewer, residence in or inclusion on a waiting list for a nursing home, or inability to communicate in English. The details of the study methods, including a map of the geographic regions employed in our analysis, are published elsewhere (20). The study was approved and monitored by institutional review boards at all participating institutions.

We used a combination of mail and telephone contact to select participants from commercially available lists of residents. We collected verbal consent and baseline data, including demographics, smoking history, cardiovascular risk factor history, and other variables, via computer-assisted telephone interview. Subsequently, an in-home visit was conducted to collect physical measurements, including blood pressure and blood and urine samples, and a signed informed consent form. These procedures were conducted on 70% of the initial telephone interview participants. Trained interviewers contact participants at 6-month intervals to assess stroke events and myocardial infarctions.

We examined the frequency of being a current smoker among race-region strata. A current smoker is determined on the basis of an affirmative response to the question, "Do you smoke cigarettes now, even occasionally?" We also examined the frequency of exposure to SHS, which was assessed only among those who replied that they were not current smokers, by the response to the question, "During the past year, about how many hours per week, on average, were you in close contact with people when they were smoking? For example, in your home, in a car, at work or other close quarters." Following the methodology of Howard et al (10), we defined exposure to SHS as being in close contact with a smoker for more than 1 hour per week; nonsmokers reporting contact of 0 hours or 1 hour per week were classified as not exposed. The main demographic factors of interest were race (African American or white), and region of residence (stroke belt, the buckle of the stroke belt, and the nonbelt area). The other factors considered in the model were age; sex; urban, rural, or mixed residence (based on census data); annual income (<$20,000, $20,000-<$35,000, $35,000-<$75,000, or ≥$75,000); and education (<high school diploma, high school diploma, some college, at least a college degree). In addition, we classified participants on self-reported residence with a smoker. Among nonsmokers, we classified participants according to their smoking history (past vs never smoker). Among those excluded from the analysis (n = 3,856) were participants for whom any data were missing.

We examined the prevalence of both current smoking and SHS exposure by race-region strata. To determine if differences in categorical variables existed across race-region strata, we used χ^2^ tests of association, and we used analysis of variance (*F* statistics) to assess whether differences across race-region strata were present among continuous variables. We employed logistic regression to assess racial and regional differences in the odds of both smoking (current vs not) and SHS exposure (exposed vs not), and how adjustment for additional nuisance sociodemographic factors affected the odds of both smoking and SHS exposure. To determine statistical significance from the logistic regression models, we used likelihood ratio χ^2^ tests. We performed sensitivity analyses to examine the effect of defining SHS exposure as 3 or more hours per week (median SHS exposure level). We used SAS version 9.1 (SAS Institute, Inc, Cary, North Carolina) for all analyses.

## Results

The cohort contained 30,229 participants, which was reduced to 26,373 by exclusion of participants with missing values for any analysis variable; the majority were excluded because they did not provide income (n = 3,740). Those with and without income data did not differ by current smoking status or SHS exposure. The remaining participants were excluded because of missing values for other covariates (n = 116).

Baseline characteristics of the study population showed that the average age differed by race-region strata (*F* = 31.7; *df* = 2; *P* < .001) (Table 1). White participants in the nonbelt region were the oldest (66.0 y, SD = 9.7 y) and African Americans in the buckle region were the youngest (62.8 y, SD = 9.0 y). Sex, urban/rural residence, income, and education all differed by race-region strata. The population in the nonbelt region had the greatest proportion of men and urban dwellers; among both African Americans and whites, nonbelt participants were wealthier and better educated than the participants of other regions. Further, the frequency of participants who reside with a smoker differed by race-region strata, and African Americans were more likely than whites to reside with a smoker, but with few regional differences. Similarly, within all regions, a higher proportion of African Americans were current smokers and were exposed to SHS than whites.

Logistic regression modeling suggested that regional differences in the odds of being a current smoker differed by race (Table 2) (χ^2^ = 27.8; *df* = 2; *P* for interaction < .001); thus, results are presented separately for African Americans and whites. The first adjusted model includes age and sex; the second also includes urban/rural residence, income, and education. Among white participants, those in both the stroke belt and the buckle of the belt were more likely to be current smokers than those in the nonbelt region in the unadjusted model; however, after adjustment for age, sex, income, education, and urban/rural residence, these relationships were no longer significant. Among African Americans, after adjustment for age and sex, the odds of current smoking for those in the stroke belt did not differ from those for residents of the nonbelt region, whereas the odds were 24% lower in the stroke buckle. Further adjustment for urban/rural living and socioeconomic factors slightly attenuated these odds ratios. After multivariable adjustment, among African Americans the odds of current smoking were significantly lower for those in the stroke belt than those in the nonbelt region, and also for those in the stroke buckle, compared with those in the nonbelt.

Similarly, logistic regression modeling indicated that regional differences in SHS exposure differed by race (χ^2^ = 8.5; *df *= 2; *P* for interaction = .01) (Table 3); thus, results are presented separately for African Americans and whites. The first adjusted model includes age and sex; the second further adjusts for urban/rural residence, income, education, and whether or not the participant was a past smoker; the final model further adjusts for whether or not the participant resides with a smoker, to determine if any observed relationships between SHS exposure and region are attributable to living with a smoker. Among African Americans, after adjustment for age and sex, the odds of being exposed to SHS were similar between residents of the stroke belt and the nonbelt regions. The odds of being exposed to SHS were 14% lower for residents of the buckle of the stroke belt compared with residents of the nonbelt region . Among whites, no differences in exposure to SHS were observed by region after controlling for age and sex. In the fully adjusted model, these relationships did not change significantly. Among African Americans, the odds of being exposed to SHS are 16% lower for residents of the buckle of the stroke belt compared with residents of the nonbelt region , again suggesting that observed regional differences remain after adjustment for urban/rural residence, income, education, smoking history, and residence with a smoker. The sensitivity analysis using the median SHS exposure time (3 h/wk) did not affect results of the analysis (data not shown).

## Discussion

For whites, rates of current smoking were slightly higher in the stroke belt and stroke buckle than in the nonstroke belt, although these associations were not significant after multivariable adjustment; thus, the higher rates in the South appear to be attributable to socioeconomic factors. In contrast, African Americans in the stroke belt and stroke buckle are less likely to be current smokers than those in the other regions after adjustment for age, sex, residence, education, and income. Regarding SHS exposure, no differences in SHS exposure by region were observed among whites, whereas African Americans in the stroke buckle may be less likely to be exposed to SHS compared with those in other regions. Hence, although SHS may be associated with risk of stroke, because the relationship between region and current smoking is not significant among whites, differences in smoking rates across regions are likely not playing a contributing role in the regional differences in stroke rates. However, for African Americans, smoking rates and exposure to SHS were in the unanticipated direction. Geographic disparities in stroke risk are larger for African Americans than for whites (14). and adjusting stroke rates for smoking and SHS exposure could result in even larger geographic disparities among African Americans. These unanticipated findings may be the result of factors such as diet or exercise that were not considered in this analysis.

Few data describe regional and racial differences in smoking habits and in SHS exposure. However, the reports published by NSDUH contrast with our findings: NSDUH found that the prevalence of current smoking differs little by race and that higher smoking rates are observed in East-South-Central US states, including Alabama, Kentucky, Mississippi, and Tennessee. A direct comparison between these regions and those employed in this study cannot be made, because the stroke belt includes Alabama, Mississippi, and Tennessee, but not Kentucky. In addition, our stroke belt also includes parts of North Carolina, South Carolina, Georgia, Arkansas, and Louisiana. State-specific estimates from the 2007 Behavioral Risk Factor Surveillance System indicate that for adults aged 18 or older, each of the states in the stroke belt (Alabama [22.5%], Arkansas [22.4%], Louisiana [22.6%], North Carolina [22.9%], South Carolina [21.9%], Mississippi [24.0%], Tennessee [24.3%]), with the exception of Georgia (19.3%), has a prevalence of current cigarette smoking higher than the national median (19.8%); however, these data are not presented by race (21). Although we also showed higher current smoking prevalence for white participants in the region that includes parts of these same states, this study failed to show higher smoking rates for African Americans, suggesting that the previously reported state-level rates could be obscuring racial differences. These differences may be a product of regional differences in smoking prevalence within states (ie, the regions in the stroke belt/buckle may have different smoking rates than other parts of the state), differences in the age distributions of the populations, differences in the period of observation, or differences in data collection methods.

These data do not support the hypothesis that current smoking and SHS exposure contribute to the racial and regional differences in stroke mortality, because adjusted rates of smoking were only slightly higher among whites in the buckle and were higher among African Americans in the nonbelt region. Further, African Americans in the buckle region had slightly less SHS exposure than those in the nonbelt region. Although several hypotheses have been proposed to explain the geographic differences in stroke mortality, including differences in gene frequency, novel risk factors, infection rates, socioeconomic status, lifestyle choices such as diet or exercise, case fatality rates, and differential causes of mortality following an initial stroke or CHD event, data to assess these potential causes are lacking (12,13).

The work described herein has some limitations. First, these data are cross-sectional; consequently, we are unable to consider the relationship between smoking and SHS exposure and subsequent risk of stroke. These analyses also rely on self-report for both current smoking status and SHS exposure, without validation by either observation or cotinine measure. However, self-reported data on current smoking status have been found to have high validity (22). In contrast, most studies suggest that biomarkers indicate higher levels of SHS exposure than those reported from questionnaires (2). However, studies have also demonstrated that people classified as having high levels of SHS exposure by self-report also had higher levels of biomarkers for SHS exposure than people who had low levels of exposure (2). Although our method of estimating SHS exposure is coarse, we believe that it will provide a dichotomy of nonsmokers who were exposed to SHS versus those who were either not exposed or were exposed to very low levels of SHS, and the measure is identical to those that have shown associations with clinical outcomes (10,13). In addition, the SHS exposure data in our study showed a similar relationship between SHS exposure and race (African Americans were more likely to be exposed than whites) to national estimates of serum cotinine among nonsmokers from 2001 through 2002 (2).

On the basis of our results, it is unlikely that either active smoking or self-reported exposure to SHS are major contributors to the observed geographic disparities in stroke mortality, although both have been implicated as factors related to overall stroke risk (2-9). However, interventions to reduce the prevalence of smoking are needed and should focus on African Americans, who, regardless of region, had higher rates of smoking than whites. In addition, substantial numbers of nonsmokers are still being exposed to SHS. The public health community needs to continue to implement laws and policies that provide completely smoke-free environments to ensure that nonsmokers are fully protected from SHS exposure (2). From 2004 through 2007, the number and restrictiveness of state laws regulating smoking in private-sector worksites, restaurants, and bars increased substantially; however, states in the South were less likely to have smoke-free laws than other states (23). Further, efforts to educate people regarding the detrimental effects of SHS exposure should also concentrate on African Americans, because African Americans are more likely to reside with a smoker and thus are more likely to be exposed to SHS.

## Figures and Tables

**Table 1:** Baseline Characteristics, Overall and by Race/Region Strata for the REGARDS Study, 2003-2007^a^

Characteristic	No. (%) n = 26,373	African American (n = 10,889)			White (n = 15,484)		

		Stroke Belt, n = 3,617	Stroke Buckle n = 1,920	Nonbelt Region n = 5,352	Stroke Belt n = 5,497	Stroke Buckle n = 3,545	Nonbelt Region n = 6,442
**Age, mean (SD), y **	65.1 (9.4)	63.2 (9.0)	62.8 (9.0)	65.5 (9.2)	65.5 (9.2)	65.2 (9.5)	66.0 (9.7)
**Female^b^**	14,122 (54)	2,247 (62)	1,242 (65)	3,145 (59)	2,697 (49)	1,929 (54)	2,862 (44)
**Residence^b^**							
Urban	18,688 (71)	2,940 (81)	1,224 (64)	4,967 (93)	3,136 (57)	1,617 (46)	4,804 (75)
Rural	5,118 (19)	456 (13)	406 (21)	347 (7)	1,576 (29)	1,176 (33)	1,157 (18)
Mixed	2,567 (8)	221 (6)	290 (15)	38 (<1)	785 (14)	752 (21)	481 (7)
**Income, $^b^**							
<20,000	5,460 (21)	1,303 (36)	611 (32)	1,438 (27)	862 (16)	496 (14)	750 (12)
20,000-<35,000	7,286 (28)	1,035 (29)	584 (30)	1,679 (31)	1,541 (28)	877 (25)	1,570 (24)
35,000-<75,000	8,880 (34)	957 (26)	572 (30)	1,604 (30)	1,949 (35)	1,380 (39)	2,418 (38)
≥75,000	4,747 (18)	322 (9)	153 (8)	631 (12)	1,145 (21)	792 (22)	1,704 (26)
**Education^b^**							
<high school diploma	3,108 (12)	757 (21)	395 (21)	863 (16)	457 (8)	315 (9)	421 (5)
High school diploma	6,738 (26)	975 (27)	559 (29)	1,461 (27)	1,454 (26)	879 (25)	1,410 (22)
Some college	7,141 (27)	898 (25)	481 (25)	1,586 (30)	1,514 (28)	964 (27)	1,698 (26)
College graduate	9,386 (36)	987 (27)	485 (25)	1,442 (27)	2,072 (38)	1,387 (39)	3,013 (47)
**Smoking status**							
Reside with smoker^b^,^c^	2,383 (11)	399 (14)	225 (14)	567 (13)	448 (9)	300 (10)	744 (8)
Current smoker^b^	3,930 (15)	674 (19)	302 (16)	958 (18)	751 (14)	503 (14)	742 (12)
Exposed to SHS^b^,^c^	3,528 (16)	590 (21)	272 (18)	802 (19)	672 (15)	452 (15)	740 (13)
Past smoker^b^	10,245 (47)	1,191 (43)	603 (39)	2,031 (49)	2,237 (48)	1,474 (50)	2,709 (48)

Abbreviations: REGARDS, Reasons for Geographic and Racial Differences in Stroke; SD, standard deviation; SHS, secondhand smoke.^a^ Data are n (%) unless otherwise noted.

a Data are n (%) unless otherwise noted.

b Indicates statistically significant tests of differences across race/region strata of sex (χ^2^ = 112.2; *df* = 2; *P* < .001), residence (χ^2^ = 1178.7; *df* = 4; *P* < .001), income (χ^2^ = 192.5; *df *= 6; *P* < .001), education (χ^2^ = 162.6; *df* = 6, *P* < .001), residence with a smoker (χ^2^ = 11.9; *df* = 2; *P* = .003), current smoking (χ^2^ = 9.2; *df* = 2; *P* = .01), exposure to SHS (χ^2^ = 8.1; *df* = 2; *P* = .02), and past smoker (χ^2^ = 12.2; *df* = 2; *P* = .002).

c Among nonsmokers.

**Table 2:** Odds of Being a Current Smoker, by Race, Among Participants in the REGARDS Study, 2003-2007

Region	African American			White		

	Univariate OR (95% CI)	Model 1^a^ OR (95% CI)	Model 2^b^ OR (95% CI)	Univariate OR (95% CI)	Model 1^a^ OR (95% CI)	Model 2^b^ OR (95% CI)
Nonbelt	1 [Reference]					
Stroke belt	1.1 (0.94-1.2)	0.95 (0.85-1.1)	0.87 (0.77-0.97)	1.2 (1.1-1.4)	1.2 (1.1-1.3)	1.1 (0.97-1.2)
Stroke buckle	0.86 (0.74-0.99)	0.76 (0.66-0.88)	0.71 (0.61-0.83)	1.3 (1.1-1.4)	1.1 (1.0-1.2)	1.2 (1.0-1.3)

Abbreviations: REGARDS, Reasons for Geographic and Racial Differences in Stroke; OR, odds ratio; CI, confidence interval.

a Adjusted for age and sex.

b Adjusted for age, sex, urban/rural residence, income, and education.

**Table 3:** Odds of Secondhand Smoke Exposure, by Race, Among Nonsmoking Participants in the REGARDS Study, 2003-2007

Region	African American				White			

	Univariate OR (95% CI)	Model 1^a^ OR (95% CI)	Model 2^b^ OR (95% CI)	Model 3^c^ OR (95% CI)	Univariate OR (95% CI)	Model 1^a^ OR (95% CI)	Model 2^b^ OR (95% CI)	Model 3^c^ OR (95% CI)
Nonbelt	1 [Reference]							
Stroke belt	1.2 (1.0-1.3)	1.1 (0.97-1.2)	1.1 (0.95-1.2)	1.1 (0.95-1.2)	1.1 (1.0-1.3)	1.1 (0.99-1.2)	1.0 (0.91-1.2)	1.0 (0.89-1.1)
Stroke buckle	0.91 (0.78- 1.1)	0.86 (0.74-1.0)	0.86 (0.73-1.0)	0.84 (0.71-0.98)	1.2 (1.0-1.3)	1.2 (1.0-1.3)	1.1 (0.96-1.3)	1.1 (0.93-1.3)

Abbreviations: REGARDS, Reasons for Geographic and Racial Differences in Stroke; OR, odds ratio; CI, confidence interval.

a Adjusted for age and sex.

b Adjusted for age, sex, urban/rural residence, income, education, and smoking history.

c Adjusted for age, sex, urban/rural residence, income, education, smoking history, and residence with a smoker.
